# The future of metacognition research: Balancing construct breadth with measurement rigor

**DOI:** 10.1016/j.cortex.2023.11.002

**Published:** 2024-02

**Authors:** Sucharit Katyal, Stephen M. Fleming

**Affiliations:** a Max Planck UCL Centre for Computational Psychiatry and Ageing Research, University College London, London, UK; b Wellcome Centre for Human Neuroimaging, University College London, London, UK; c Department of Experimental Psychology, University College London, London, UK

**Keywords:** Metacognition, Measurement, Self-knowledge, Confidence

## Abstract

Foundational work in the psychology of metacognition identified a distinction between metacognitive knowledge (stable beliefs about one’s capacities) and metacognitive experiences (local evaluations of performance). More recently, the field has focused on developing tasks and metrics that seek to identify metacognitive capacities from momentary estimates of confidence in performance, and providing precise computational accounts of metacognitive failure. However, this notable progress in formalising models of metacognitive judgments may come at a cost of ignoring broader elements of the psychology of metacognition – such as how stable meta-knowledge is formed, how social cognition and metacognition interact, and how we evaluate affective states that do not have an obvious ground truth. We propose that construct breadth in metacognition research can be restored while maintaining rigour in measurement, and highlight promising avenues for expanding the scope of metacognition research. Such a research programme is well placed to recapture qualitative features of metacognitive knowledge and experience while maintaining the psychophysical rigor that characterises modern research on confidence and performance monitoring.

## 1 Introduction

Metacognition refers to the capacity to reflect on, evaluate and control first-order cognitive processes such as decision-making, memory and perception. Accurate metacognition – often assayed as the extent to which subjective confidence tracks objective performance – is considered foundational to flexible, adaptive behaviour in a range of settings, with dysfunctional metacognition linked to detrimental outcomes in educational and clinical settings, and in social coordination. Research on the neuroscience of metacognition has gained considerable pace in recent years, with growing insights into the subpersonal mechanisms that contribute to self-evaluation. A key focus here has been on the formation of confidence (or, conversely, the recognition of error) as a canonical metacognitive operation that tracks first-order performance. For instance, in the 1980s, pioneering neuropsychological studies suggested that patients’ metacognition about their performance in simple memory tasks may be impaired by brain lesions that leave memory performance itself intact – suggesting a specific neural basis for metacognitive capacity (Janowsky, Shimamura, & Squire, 1989; Shimamura & Squire, 1986). And since the early 2000s, with the advent of both functional neuroimaging and animal models of confidence, there has been an explosion of interest in neural and computational processes involved in metacognition and performance monitoring (for reviews see Rouault, McWilliams, Allen, & Fleming, 2018; Fleming & Dolan, 2012; Kepecs & Mainen, 2012; Meyniel, Sigman, & Mainen, 2015).

Our goal here is not to review this burgeoning literature. Instead, we offer a critical perspective, suggesting that the pursuit of a rigorous neuroscience of metacognition, while of foundational importance, may have inadvertently discarded some of the more interesting aspects of the original construct. We first provide a brief historical perspective on the measurement of metacognition, highlighting how advances in measurement led to new neuroscientific findings, before critically evaluating whether measurement rigor may have come at the cost of a narrowing of the questions we seek to ask within metacognitive neuroscience. We close by proposing ways to recapture qualitative features of metacognitive knowledge and experience that were part of the original psychological construct, while maintaining the psychophysical rigor that characterises modern research on confidence and performance monitoring.

## 2 The scope of metacognition research

The study of metacognition gained prominence in the 1970s and 80s under the umbrella of work in development, educational psychology and neuropsychology (for reviews see Flavell, 1979; Metcalfe, D. of P. J, Metcalfe, & Shimamura, 1994; Nelson & Narens, 1990), following the recognition that children’s self-assessments of their own abilities were important in guiding learning, although often not as accurate as the same assessments made by adults (Flavell, 1979). For instance, at the start of his famous 1979 paper, “Metacognition and Cognitive Monitoring: A new area of cognitive-developmental inquiry”, Flavell describes the following classroom situation: “… older subjects studied for a while, said they were ready, and usually were, that is, they showed perfect recall. The younger children studied for a while, said they were ready, and usually were not”. A core feature of metacognition, then, is that it encompasses subjects' beliefs about an ongoing performance episode – with the implication that such beliefs are important for shaping what people do next.

Conceived in this manner, metacognition represents a broad feature of human mental life that supplements a range of first-order cognitive processes. Such a perspective suggests that accurate metacognition should come along with widespread functional benefits (Nelson & Narens, 1990). For example, when preparing for an exam on a subject, the amount of time and effort a student puts in is guided by (among other things) their beliefs about how well-versed they are with that subject, and their ability to retain information in memory. Conversely, if they mistakenly think they have studied sufficiently well, they might go into the exam with misplaced confidence, and fail – even if their raw aptitude for the subject is adequate. Accordingly, recent research has highlighted a delicate interplay between the accuracy of metacognitive operations and success on tests of fluid intelligence (Bocanegra, Poletiek, Ftitache, & Clark, 2019; Bulley & Schacter, 2020; Fandakova et al., 2017).

Flavell (1979) went on to propose a distinction between metacognitive knowledge (or metacognitive beliefs) – “everything you could come to believe about the nature of yourself and other people as cognitive processors” – and metacognitive experience – online feelings or other conscious experiences about one’s cognitive processes. Metacognitive knowledge was further proposed to distinguish between personal factors (e.g., believing that I am better at tennis than my brother), and task factors (believing that I am better at tennis than I am at golf). Flavell also proposed a delicate interplay between knowledge and experience – for instance, in the middle of a physics exam, I might experience disfluency or lack of confidence in answering a particular question, leading me to update my beliefs (knowledge) about my aptitude for studying physics, and in turn reducing the likelihood I will choose to study physics again in the future (a form of metacognitive control). In the following sections, we focus on metacognitive evaluation, which broadly encompasses metacognitive knowledge and metacognitive experiences, and for which empirical measures have developed apace in recent years. We do not consider metacognitive control – the role of metacognitive evaluation in the guidance of behaviour – despite this being an equally important topic of study within the broader field of metacognition research.

## 3 A brief history of metacognitive measurement

A natural method for eliciting metacognitive judgments is via self-report questionnaires. Such methods assay global beliefs about one’s performance capacities – for instance, the use of the Metamemory in Adulthood (MIA) or Memory Functioning Questionnaire (MFQ) for recording subjects' beliefs about their memory capacity (Dixon, Hultsch, & Hertzog, 1988; Gilewski, Zelinski, & Schaie, 1990). However, self-report assays of metacognitive capacity itself – the second-order property of whether one’s metacognitive assessments track performance – are on shaky ground, precisely because self-report questionnaires presuppose the metacognitive awareness of mental function that they seek to measure. For example, the MIA includes questions such as “How is your memory compared to what it was one year ago?” When responding to such questions, we would expect high estimates of one’s current memory not only from someone with good memory and accurate metacognition, but also potentially from someone with poor memory and poor metacognition, because by definition, the latter are unable to accurately assess their low memory capacity. An alternative approach therefore is to compare one-shot judgments of one’s performance with a measure of actual performance (or a care-giver rating of such performance in clinical investigations). However, such discrepancy scores are unable to distinguish between bias in estimation and sensitivity to performance (Fleming & Lau, 2014). In other words, if someone substantially overestimates their memory capacity, it is unclear if they have poor metacognitive insight or if they have a general tendency to use high ratings. Instead, for assessing metacognitive capacity, indirect, task-based methods are required where first-order performance is both measured and accounted for.

Task-based quantification of metacognition was initially pursued in research on metamemory, which pioneered the use of rating procedures to assess, over many trials, how people’s metacognitive judgments (such as confidence ratings, and feelings of knowing), related to their first-order performance (Clarke, Birdsall, & Tanner, 1959; Hart, 1965) (other research in the psychophysics tradition studied task-based confidence much earlier than this, although without considering it as a window onto metacognition; Henmon, 1911; Peirce & Jastrow, 1884). In these studies, participants are required to evaluate their performance multiple times during the course of the experiment, allowing a statistical picture to be formed of how variation in self-evaluation (low vs. high confidence) relates to objective performance. As Nelson and Narens write, “… people are construed as imperfect measuring devices of their own internal processes” (Nelson & Narens, 1990). Using these methods, it is possible to quantify the accuracy of a number of different flavours of metacognitive judgment – feelings of knowing (FOKs), prospective and retrospective judgments of learning (JOLs), retrospective confidence judgments in first-order decisions, and so on. It was subsequently recognised that many of these judgment types can be (computationally) formulated as retrospective or prospective judgments of confidence in another cognitive process (Fleming & Dolan, 2012; Kepecs & Mainen, 2012; Meyniel et al., 2015; Pouget, Drugowitsch, & Kepecs, 2016; Yeung & Summerfield, 2012) – and thus confidence became a core variable of interest for metacognition research.

The stage was then set for a powerful marriage of confidence-based approaches to metacognition and detailed, performance-controlled approaches derived from psychophysics. Due to the focus of psychophysics on vision research, this led to a new field of visual metacognition (Mamassian, 2016; Rahnev et al., 2022)– although the methods that were developed are applicable more widely, and are now gaining traction in other domains such as audition, olfaction, touch, interoception, memory, decision-making and so on (De Martino, Fleming, Garrett, & Dolan, 2013; Faivre, Filevich, Solovey, Kühn, & Blanke, 2018; Gardelle, Corre, & Mamassian, 2016; Legrand et al., 2022; Harrison et al., 2021; Jönsson & Olsson, 2003). The important point for our current purposes is that new frameworks were rapidly developed to characterise the statistical properties of confidence judgments, and how they relate to objective performance.

A central challenge in this endeavour is how to ensure metrics of metacognition are “pure” and uncontaminated by other confounding influences. This is particularly tricky in metacognition research, because metacognition is itself influenced by an (imperfectly controlled) first-order cognitive process (Peters, 2022). This means that secure inference on metacognitive processes requires not only controlling stimulus input (as would be done in an experiment on perception, or learning, for instance), but also appropriately controlling or modelling variation in first-order performance. The pursuit of more precise control over performance confounds characterises much of the methodological development in the field over the past 15 years.

Initial task-based approaches to quantifying metacognitive capacity relied on correlation measures like *phi* – the standard Pearson correlation between accuracy and confidence – and the Goodman-Kruskall gamma coefficient (Goodman & Kruskal, 1979; Nelson, 1984) to assess the link between trial-by-trial performance and confidence. The advantage of these correlation measures is that they can be applied to any task. Such measures however suffer from conflating metacognitive ability (hereon, metacognitive sensitivity) with changes in either first-order performance or metacognitive bias – the tendency to use higher or lower confidence ratings on average (Fleming & Lau, 2014; Masson & Rotello, 2009). An advance beyond correlational measures was the adoption of receiver operating characteristic (ROC)-based methods inspired by signal detection theory (SDT). Just as the area under a standard (type 1) ROC curve (AUROC) characterises the extent to which subjects' responses discriminate two or more world states (e.g., stimulus presence vs. absence) irrespective of criterion placement, the area under the type 2 ROC (AUROC2) characterises the extent to which confidence discriminates between correct and incorrect trials irrespective of confidence criterion placement (Clarke et al., 1959; Galvin, Podd, Drga, & Whitmore, 2003). AUROC2 therefore provides a compact, bias-free summary – a single number – that indexes a subject’s metacognitive sensitivity. However, while AUROC2 is independent of metacognitive bias, it remains sensitive to changes in first-order performance. Thus, when using AUROC2 as a measure of metacognition, care must be taken to carefully match performance between conditions or subjects (Fleming, Weil, Nagy, Dolan, & Rees, 2010; Song et al., 2011).

A major advance in deriving a pure measure of metacognitive sensitivity was the development of the meta-*d*’ model by Maniscalco and Lau (Maniscalco & Lau, 2012). This model seeks to identify the best-fitting sensitivity parameter that characterises an individual’s AUROC2 within a signal detection theory framework. Because this parameter is fit to observers' confidence ratings, rather than their first-order performance, it is denoted as meta-*d*’. Greater AUROC2 values are associated with higher meta-*d*’ values. The elegance of the approach is that meta-*d*’ is in the same units as observed first-order performance (*d*’), and thus a performance-controlled metric of metacognitive capacity, known as metacognitive efficiency, can be derived as the ratio between these two parameters (meta-*d*’/*d*’), often referred to as *Mratio.* For this reason, *Mratio* is considered a gold-standard metric and has been widely used in empirical studies, including in identifying neural correlates of metacognition (e.g., Fleming, Ryu, Golfinos, & Blackmon, 2014; McCurdy et al., 2013; Shekhar & Rahnev, 2018; Ye, Zou, Lau, Hu, & Kwok, 2018; Zheng et al., 2021), studying the domain generality of metacognitive efficiency (e.g., Fitzgerald, Arvaneh, & Dockree, 2017; Mazancieux, Fleming, Souchay, & Moulin, 2020; Morales, Lau, & Fleming, 2018) and quantifying the effects of metacognitive training (e.g., Carpenter et al., 2019; Rouy et al., 2022). Recent hierarchical versions of the meta-*d’* model moreover allow more accurate group-level inference in situations with limited data available per subject, such as in clinical studies (Fleming, 2017).

Refining these metrics and models is still ongoing. The assumption that *Mratio* is independent of metacognitive biases (average confidence) has been recently challenged by studies showing that using higher levels of confidence ratings can lead to inflated values of *Mratio* (Shekhar & Rahnev, 2021b; Xue, Shekhar, & Rahnev, 2021). Similarly, the assumption that *Mratio* is performance-independent has been systematically evaluated in both simulation and empirical studies, with nonlinearities in this relationship leading to new model-based metrics with more stable psychometric properties (Barrett, Dienes, & Seth, 2013; Guggenmos, 2021, 2022). Another issue that has come to the fore with several metacognitive measures including *Mratio* is that staircasing procedures commonly used to control first-order performance can artificially inflate metacognitive efficiency (Rahnev & Fleming, 2019). This is because the variation in task difficulty introduced by the staircase can itself be used as a cue to confidence (more difficult trials are less likely to be correct), thus obscuring inference on endogenous metacognitive efficiency.

Another issue is that the meta-*d*’ framework is not a process model of how confidence ratings are generated (Shekhar & Rahnev, 2021b), and thus cannot identify distinct sources of metacognitive inefficiency (Shekhar & Rahnev, 2021a). Thus, just as vision scientists may investigate the different component processes that lead to a particular *d*’, metacognition researchers are increasingly turning to richer computational models to decompose the different stages involved in confidence formation (Bang & Fleming, 2018; Boundy-Singer, Ziemba, & Goris, 2022; Guggenmos, 2022; Shekhar & Rahnev, 2018). Of particular interest here is whether confidence reflects a heuristic such as distance to a decision criterion or bound (Kepecs, Uchida, Zariwala, & Mainen, 2008; Vickers, 1979), or whether it is Bayesian or quasi-Bayesian in also being sensitive to sensory uncertainty (Adler & Ma, 2018; Aitchison & Lengyel, 2017; Denison, Adler, Carrasco, & Ma, 2018; Li & Ma, 2020). It is beyond the scope of the current paper to review this literature, but we note one promising way forward here is to consider metacognitive capacity (and summary statistics such as meta-d') as resulting from the fidelity of a number of different processing stages, including sensitivity to perceptual or evidential uncertainty (Boundy-Singer et al., 2022; Geurts, Cooke, van Bergen, & Jehee, 2022), frame-of-reference shifts needed to monitor one’s own response (Bang & Fleming, 2018; Desender, Ridderinkhof, & Murphy, 2021; Fleming & Daw, 2017), and finally the requirement to explicitly represent or use a metacognitive estimate in communication and behavioural control (Bang, Ershadmanesh, Nili, & Fleming, 2020; Donoso, Collins, & Koechlin, 2014; Shekhar & Rahnev, 2018). Another promising avenue of research is to ask how the formation of local confidence unfolds over time, and how changes in global priors that might affect this local confidence accumulation process (Desender, Vermeylen, & Verguts, 2022; Marcke, Denmat, Verguts, & Desender, 2022; Pleskac & Busemeyer, 2010). Unpacking these processing stages, and providing a more detailed computational account of metacognition, remains a major goal for the field (Rahnev et al., 2022).

## 4 Construct breadth in metacognitive neuroscience

The brief historical review in the previous section showcases how the field of metacognition research has become increasingly secure in deriving a relatively “pure” index of metacognitive capacity from confidence in behavioural reports, one that is now driving forward new process models of how such a capacity is underpinned at computational and neural levels. This is an impressive achievement, based on rapid progress made within the past 15 years.

We wholeheartedly endorse this progress, and are invested in developing the methods and models described above. However, we also urge that, in the general enthusiasm to dig deeper, we should take care that the well that is dug does not become too narrow. As the quantification of metacognition has become more refined, there is a danger that some of the varieties and functions of metacognition originally highlighted in the social and developmental psychology literatures becomes lost. A related concern is that when a psychological construct becomes operationalised within a task or metric, such as confidence-in-performance, this then ushers in a science of the task or metric, rather than of the construct. A number of problems may ensue as a result. One is opportunity cost – researchers may spend time and money in pursuing ever-more detailed models of confidence while neglecting other under-researched aspects of metacognition. Another is conceptual slippage – we might apply models and metrics such as meta-d’ to measure other aspects of metacognition that are not appropriately tracked by these metrics. More broadly, continuing to plough the furrow offered by precise and well-defined measures of one aspect of metacognition may lead theories of metacognition to become myopic or biased, such that the external validity of metacognition research may suffer. We are not suggesting throwing away the progress that has been made on models of confidence formation, and we provide a spirited defence of their usage below. But we also argue that much of the richness of human metacognition is currently untapped by current methods, leading to new opportunities for research.

Why is confidence (and indices of the sensitivity of confidence ratings such as meta-*d’*) such an important variable of interest in metacognition research? A simple answer is that confidence (or uncertainty) is a second-order property that indexes one’s doubt or certainty in another (first-order) quantity. Such doubt often refers to external events – for instance, I can be more or less confident in Manchester United winning the Premier League, or in interest rates rising this year. But when confidence refers to one’s own cognitive or physical actions, it becomes self-referential, and a measure of self-doubt. As Peter Carruthers describes:

*“Suppose that I judge that the longest among nine lines on a screen in front of me is the one on the left, but I also judge that I am uncertain. This isn’t the same as attributing ignorance that the one on the left is the longest, obviously, since I am currently judging that it is. Rather, I would seem to be judging of my judgment that there is a significant chance that it is mistaken.”* (Carruthers, 2011, p. 283)

An explicit judgment of confidence about one’s own behavioural performance is therefore a canonical metacognitive operation – a judging of one’s own judgment. Its fidelity with respect to task performance – metacognitive sensitivity – is therefore also a useful index of metacognitive capacity, as it tracks to what extent such judgments are being informed by task and skill-relevant information.

**Fig. 1:**
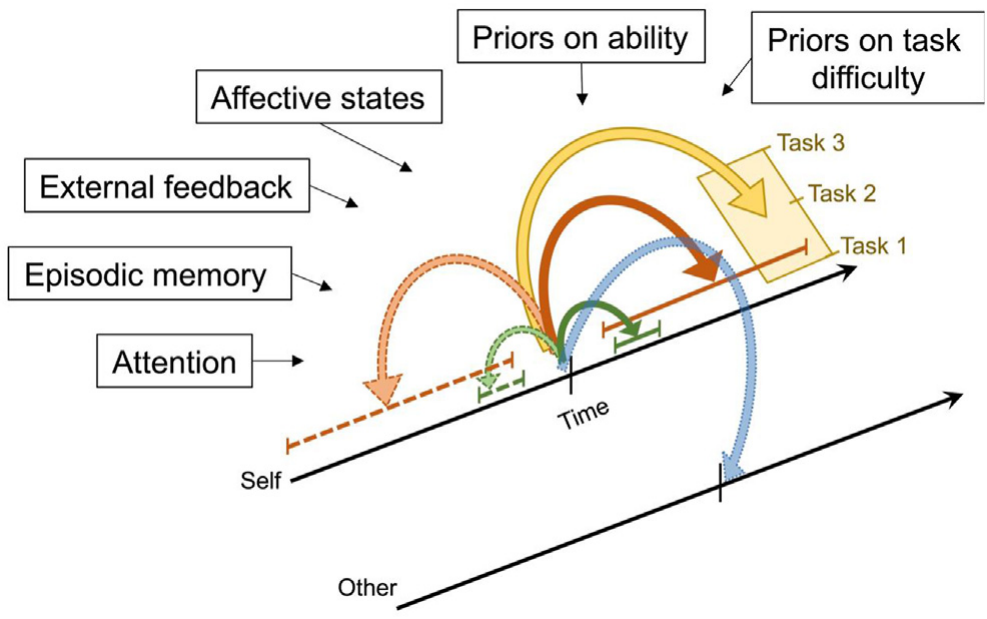
**The breadth of metacognition.** At a given time, metacognitive evaluations can be made prospectively (coloured arrows, solid) or retrospectively (dashed). They can also be made about local decisions (i.e., for a particular instance of a task; green arrows) or globally, integrating over a larger timespan (orange arrows). Metacognitive judgments may also integrate over a number of different tasks or domains. Metacognitive evaluations can also be made for other individuals (blue arrow) for all possible combinations of timespans and domains. Finally, a number of factors may influence these self- and other-evaluations (text boxes).

This is the positive case for operationalising the construct of metacognition as confidence and utilising metrics like meta-*d*’ for assessing metacognitive capacity. However, this approach is blind to a large swathe of metacognitive processing, particularly that which underpins the formation of metacognitive judgments over longer timescales (Fig. 1), and where the target first-order processes do not have obvious truth or correctness conditions observable in behaviour (such as metacognitive judgments of affective states). In what follows, we suggest approaches to redressing this balance.

## 5 Expanding the breadth of metacognition research

### 5.1 Local and global metacognition

Most confidence research has focused on “local” judgments of performance on individual trials or instances of a task. In contrast, a distinct literature in social psychology and judgment and decision-making research has asked how people evaluate themselves on a more global level – asking, for instance, how they would rank their driving or intellectual abilities (Bandura, 1977; Dunning, Heath, & Suls, 2004). These global estimates are self-beliefs referring to performance over longer timescales, and more akin to Flavell’s metacognitive knowledge. Currently, the development of frameworks and toolkits for the study of metacognitive knowledge has lagged behind. We suggest that such development remain tightly integrated with the progress that has been made on understanding confidence – as “local” metacognitive experiences likely inform and shape our rich metacognitive knowledge base.

Recently Seow, Rouault, Gillan and Fleming (2021) proposed that these two levels – “local” and “global” metacognition – should not be viewed as separate, but instead can be conceived of as a hierarchy, with potentially bidirectional interactions. For example, a student may feel confident in a particular answer on a test (a local metacognitive judgment), which affects their estimate of performance across the whole exam (a global judgment), which in turn affects their estimate of their academic aptitude (an even more global judgment).

One finding commonly attributed to a deficit in global metacognition is the Dunning-Kruger effect (Kruger & Dunning, 1999), in which poor performers tend to overestimate their performance when asked to give one-shot ratings in a number of different domains. This coupling of global miscalibration to low performance is often explained by worse performers lacking the skills needed to effectively judge local performance fluctuations. Recently, this hypothesis has been tested using computational approaches that relate local confidence formation to global ratings of performance (Jansen, Rafferty, & Griffiths, 2021; McIntosh, Moore, Liu, & Della Sala, 2022). Jansen et al. (2021) developed a model in which rational subjects had access to a noisy representation of response accuracy. In a Bayesian framework, due to regression of performance estimates to a prior mean, low performers already appear to overestimate their performance – producing a Dunning-Kruger effect without any metacognitive deficit. However, Jansen et al. also documented subtle nonlinearities in the relationship between performance and metacognitive noise in the tails of the distribution in a large online sample – suggesting an additional contribution of local metacognition. Adopting a task-based approach, and measuring local metacognitive efficiency, McIntosh et al. (2022) similarly found that while metacognitive differences can contribute to the Dunning-Kruger effect, they are neither necessary nor sufficient for producing it.

Recently, novel laboratory tasks have been designed to study interactions between local and global confidence (Cavalan, Vergnaud, & de Gardelle, 2023; Lee, de Gardelle, & Mamassian, 2021; Rouault, Dayan, & Fleming, 2019). Rouault et al. discovered that fluctuations in local confidence during a perceptual task explained end-of-block global judgments (Rouault et al., 2019). Notably, local confidence was both a necessary and sufficient predictor of global judgments, as after accounting for confidence, local changes in accuracy or response time no longer significantly predicted global judgments. Using fMRI, Rouault and Fleming (2020) used a similar local-global confidence paradigm to reveal that ventral striatal activity reflected the level of global self-beliefs (but not local confidence signals) while local confidence-related activity in ventromedial PFC (vmPFC) was further modulated by the level of global self-belief. This work is also in line with other studies that have identified a role for the vmPFC in integrating local confidence over longer timescales to form aggregate self-performance estimates (Wittmann et al., 2016). Together these studies indicate a neuroanatomical nexus where local and global confidence signals interact.

Other work has identified intriguing disconnections between local and global metacognition, particularly in relation to the transdiagnostic psychiatric symptom dimension of compulsive behaviour. Hoven, Luigjes, Denys, Rouault, & van Holst (2023) found that while the degree of compulsivity was positively related to local confidence – replicating previous work (Rouault, Seow, Gillan and Fleming, 2018) – it was negatively related to global confidence. The negative association of compulsivity and global confidence is consistent with a large body of work showing that obsessive-compulsive disorder is characterised by underconfidence (for review see, Hoven et al., 2019), suggesting that mental health symptoms may be differentially related to distinct aspects of metacognition.

In addition to being extended in time, higher levels of a metacognitive hierarchy may also have a wider scope in terms of integrating over multiple cognitive processes/abilities. In other words, towards the top of the hierarchy, confidence estimates can integrate across increasingly diverse inputs from different sensory modalities. This may result in global self-beliefs being influenced by processes unfolding across multiple task domains – leading, for instance, to changes in interoceptive processing (or precision) impacting upon our (global) confidence about other domains of perception and cognition (Allen et al., 2016; Stephan et al., 2016). At the same time, shifts in global self-beliefs may also mediate “leaks” in confidence between tasks (Rahnev, Koizumi, McCurdy, D’Esposito, & Lau, 2015). At even higher levels of a hierarchy, broad, domain-agnostic self-beliefs may modulate feelings of self-esteem or self-worth (Rouault, Will, Fleming, & Dolan, 2022).

This work on local and global metacognition suggests that metacognitive experiences and metacognitive knowledge may not be entirely distinct constructs, as also originally noted by Flavell. Instead, there may be a continuum in which increasingly stable self-beliefs (metacognitive knowledge) are formed by integrating local confidence over increasingly longer timescales. Maintaining beliefs at different timescales is a natural consequence of hierarchical predictive processing schemes, where higher levels of the hierarchy furnish slower-evolving priors on faster processes unfolding lower down the hierarchy (those which are more immediately coupled to the sensorium). Under such schemes, the precision or confidence in beliefs at each level also needs to be estimated, to control the relative balance between top-down and bottom-up influences (Yon & Frith, 2021). An attractive hypothesis is that higher-level precision estimates furnish global self-beliefs, as they index our confidence in subpersonal processes such as motor skill or perceptual acuity. A precise mechanistic and computational model of how the different levels of a putative metacognitive hierarchy are related to each other is yet to be established. As a step towards this goal, Rouault et al. (2019) modelled global self-estimates of performance as the probabilistic combination of multiple instances of local confidence and performance feedback. According to such a model, differences between global self-estimates of performance and true performance arise from uncertainty due to the lack of a sufficient number of local task instances. A consequence is that such estimates should become more precise as local task experience increases.

Such models overcome the limitation of circularity in self-report measures, as here global metacognitive ability is estimated as the uncertainty in self-estimation relative to ground truth (aggregate) performance (Cavalan et al., 2023; Katyal, Huys, Dolan, & Fleming, 2023; Lee et al., 2021; Rouault et al., 2019). These models can moreover be extended to account for various kinds of biases/distortions in the formation of global metacognition. For example, we recently extended this model to study how global underconfidence is maintained in individuals with transdiagnostic anxiety and depression symptoms (Hoven et al., 2023; Rouault, Seow, et al., 2018). By manipulating performance feedback, we tested whether global underconfidence resulted from a) greater sensitivity to negative compared to positive feedback, b) greater sensitivity to low compared to high local confidence, and/or c) a general negative response bias when reporting confidence (Katyal et al., 2023). We found that individuals with high anxiety and depression symptoms were more sensitive to instances of low (compared to high) local confidence when forming their global confidence judgments, despite intact sensitivity to feedback valence. In other words, anxious-depressive symptomatology tracked distortions in the interaction between local and global metacognition. Further extrapolating such a model to consider interactions between different levels of a putative metacognitive hierarchy (for example, combining across tasks) may facilitate a computational account of distortions in domain-general self-beliefs that have been associated with personality and mental health traits.

At the same time, there are likely to be several other influences on global metacognitive judgments that are yet to be explored, and that would augment such a model. Some guiding principles here can be derived from the literature on self-efficacy, which has identified personal experiences of success, vicarious social experiences, physiological and emotional state, and motivational persuasion as key influences on self-efficacy formation (Bandura, 1977). For instance, it remains unknown how local confidence and explicit feedback interact to shape global judgments (Rouault et al., 2019), or whether episodic memories of salient successes or failures influence the formation and maintenance of global self-beliefs – analogous to the role of episodic memory in learning about rewards (Bornstein, Khaw, Shohamy, & Daw, 2017; Rosenbaum, Grassie, & Hartley, 2022). In turn, because global metacognitive estimates integrate over longer timescales, it is likely that contextual factors such as attention or emotional state modulate the degree to which local confidence is integrated into global self-beliefs. Finally, a prominent source of global self-beliefs may be observing similar others perform the same task, to allow a prior to be developed about our own likely chance of success. Understanding this social aspect of global metacognition will benefit from a more detailed understanding of how we infer confidence in the decisions of others (Bang, Moran, Daw, & Fleming, 2022; Boorman, O’Doherty, Adolphs, & Rangel, 2013; Patel, Fleming, & Kilner, 2012; Trudel, Rushworth, & Wittmann, 2021; Wittmann et al., 2016).

More generally, understanding global metacognition may have relevance for applied aspects of metacognition research, for instance, in education (Fleur, Bredeweg, & van den Bos, 2021). For example, global metacognition about how well one understands a topic or a subject may be a key driver of the investment of study time (Nelson & Narens, 1990).

### 5.2 Symmetries between self- and other-evaluation

Another attractive avenue for the study of broader facets of metacognitive knowledge is examining symmetries (or asymmetries) between processes involved in constructing self- and other-knowledge. A rich tradition in social psychology has asked how people represent the traits and mental states of others (Baron-Cohen, 1991; Gallagher & Frith, 2003). It has often been suggested that self-directed metacognition relies in part on theory-of-mind abilities that are in the business of maintaining and updating knowledge about others (Carruthers, 2009, 2011; Vaccaro & Fleming, 2018). There is indirect evidence for this view from developmental studies that find the ability to explicitly monitor self-performance using confidence ratings is gained around the same age (4-5 years old) as children begin to pass tests of theory-of-mind ability (Hembacher & Ghetti, 2014; Lockl & Schneider, 2007). Recent studies have also found that subjects with Autism Spectrum Disorder (ASD) show impairments both in measures of mentalising about others, and of explicit self-directed metacognitive efficiency (Johnstone, Friston, Rees, & Lawson, 2022; Nicholson, Williams, Lind, Grainger, & Carruthers, 2021; van der Plas et al., 2021); although see Embon, Cukier, Iorio, Barttfeld, and Solovey (2022)). For example, in a dual-task scenario, a mentalising task (but not a similarly demanding non-mentalising task) impairs the fidelity of (self-directed) confidence ratings on a metacognition task, indicating a sharing of cognitive resources between self-directed metacognition and mentalising about others (Nicholson et al., 2021).

So far, these studies have used off-the-shelf metrics of mindreading and metacognitive efficiency (i.e., measures developed to study the two processes in isolation), with limited attempt to relate the shared computations underpinning self- and other-directed processes (although see Bang et al., 2022; Patel et al., 2012; Trudel et al., 2021). A profitable avenue of research, then, would be to consider how we build both local and global metacognitive estimates of our own and others' performance across a number of distinct domains. It is likely that the formation of local confidence judgments relies on direct access to a number of private cues – such as representations of stimulus uncertainty, response fluency, and so forth – that are unavailable when judging others, and therefore the mechanisms of local confidence formation might be largely distinct for self and other (Bang et al., 2022). However, a subset of cues such as response times may be publicly observable, and in these cases shared processes may contribute to metacognition about self and other (Patel et al., 2012).

### 5.3 Affective metacognition

Currently, most research on metacognition – including the extensions we have suggested above – focuses on first-order cognitive processes that can be verified against objective performance measures. But much of human metacognition likely involves reflecting on processes that do not have an obvious ground truth – i.e., where “correctness” of metacognitive evaluation cannot be referenced against an objectively measurable first-order state (such as task performance). This is the case, for example, when estimating our confidence in subjective, value-based decisions (De Martino et al., 2013; Lebreton, Abitbol, Daunizeau, & Pessiglione, 2015), aesthetic judgments (Skov & Nadal, 2020), or one’s affective state more generally (e.g., an individual may report feeling sad, but on some occasions be very certain they are sad and other times not so certain). Here, in the absence of an objective ground truth, the “accuracy” of metacognition may be reflected by the self-consistency (Koriat, 2012) or reliability (De Martino et al., 2013) of the metacognitive evaluation with regards to a first-order valuation or affective state.

A few studies have made progress towards understanding metacognition of subjective states. De Martino et al. (2013) asked hungry participants to choose their preference between two snack items and rate their confidence in the judgment. The subjective value of these items was then measured separately by having participants provide a bid price for each snack. People’s choices were more closely informed by the subjective value difference of the two items on high-confidence trials compared to low-confidence ones, revealing that metacognitive judgments systematically tracked subjective choice consistency. Both confidence and subjective value were correlated with vmPFC activation, whereas confidence (but not value) was correlated with activity in lateral frontopolar cortex – drawing a link between the neural basis of confidence in subjective value, and prefrontal networks supporting metacognition in other performance domains (Rouault, McWilliams, et al., 2018). Another study highlighted how confidence is quadratically related to subjective ratings (Lebreton et al., 2015). In other words, intermediate ratings are accompanied by lower confidence, on average, compared to the higher and lower extremes of the scale. This effect was found across a range of estimated quantities (age, pleasantness, probability) and is consistent with a normative model of how uncertainty manifests in subjective ratings that are mapped to a linear scale. The same study also found signatures of both subjective value and its associated confidence in the vmPFC.

Similar methods may prove useful for studying metacognition of affective states. A number of studies have investigated whether people’s global assessments of the capacity to recognise others' emotions (such as self-ratings of empathy) predict objective performance on tasks of emotion recognition (for reviews see Ickes, 1993; Kelly & Metcalfe, 2011). The general conclusion from this work is that people have relatively poor (global) metacognitive estimates of their ability to recognise others' emotions, though such ratings suffer from issues highlighted above in conflating metacognitive sensitivity and bias. More recently, Kelly and Metcalfe (2011) found that trial-by-trial fluctuations in confidence predict performance on an emotional recognition task, suggesting local rather than global metacognition may be more sensitive to emotion recognition performance. However, the capacity to assign a precision or confidence level to one’s own affective states is less well explored – likely due to the challenge associated with devising experimental tools to dissociate metacognition (confidence) from first-order sensitivity in this domain. Unlike emotion recognition in others, which can be quantified using external stimuli designed to signal a particular emotional state, the measurement of objective markers of dynamically changing affective states within the same individual is conceptually and methodologically fraught.

One promising avenue for isolating confidence in affective states is via adaptation of the methods used to study confidence in value-based judgments (De Martino et al., 2013). For instance, if a subjective ground truth can be established via behavioural or subjective markers of emotional states, then one could assay people’s ability to distinguish between these states (assaying first-order sensitivity) and probe their confidence in such discrimination (assaying metacognitive sensitivity). Alternatively, implicit measures of precision (confidence) in self-evaluating one’s affective states could be extracted by applying normative computational models to the profile and response times of subjective ratings (Lebreton et al., 2015).

Affective metacognition may play an important role, for example, in emotion regulation (McRae & Gross, 2020) or be a key mechanism mediating metacognitively oriented therapeutic interventions (Moritz & Woodward, 2007; Wells, 2011). More generally, this avenue of research could also address questions concerning whether a putative domain-generality of metacognition generalises to encompass affective states (i.e., if having good metacognition about one emotional state also predicts good metacognition about other emotional states), whether affective metacognition can be trained, and whether and how it is related to interoceptive states (Garfinkel & Critchley, 2016; L. F. Barrett & Simmons, 2015; Seth, 2013), mental health, and clinical insight (David, Bedford, Wiffen, & Gilleen, 2012). There are also other scenarios besides emotion judgments where metacognitive evaluation may lack an obvious ground truth, but is nevertheless amenable to empirical investigation – such as metacognition about mental imagery, motor intentions, or pain (Arbuzova et al., 2021; Beck, Peña-Vivas, Fleming, & Haggard, 2019; Pearson, Rademaker, & Tong, 2011).

## 6 Conclusions

Much progress has been made in recent decades in understanding the statistical properties of confidence judgments about local decisions on a range of tasks. However, this pursuit of measurement rigour in the study of metacognition may be leading to a narrowing of the original construct, such that many of its salient aspects – notably the interplay between metacognitive knowledge and metacognitive experience – remain poorly understood. We suggest ways in which the construct of metacognition can be re-expanded while maintaining methodological rigour. Promising recent work has begun in this direction through the study of how global metacognitive knowledge is formed, and how links between local and global metacognition are related to changes in mental health. Finally, a broader understanding of metacognition will also benefit from a greater integration between social psychology and computational neuroscience – facilitating the development of rich frameworks that accommodate distinctions between self- and other-directed metacognition, and self-evaluations that go beyond performance or skill to also encompass affective states.
